# Following the Pandemic: Exploring Long COVID’s impact on Global Health through the World Heart Federation Global COVID-19 Study

**DOI:** 10.5334/gh.1468

**Published:** 2025-09-12

**Authors:** Karla Santo, Leandro Favaro, Eduardo Martins

**Affiliations:** 1 Hospital Israelita Albert Einstein, São Paulo, SP, Brazil; 2 Universidade Federal de São Paulo (UNIFESP), São Paulo, SP, Brazil; 3 Instituto do Coração (InCor), Hospital das Clínicas HCFMUSP, Faculdade de Medicina, Universidade de São Paulo, São Paulo, SP, Brazil

**Keywords:** COVID-19, global health, mortality, morbidity, long-term outcomes

## Abstract

Although the COVID-19 pandemic crisis has come to an end, Long COVID continues to pose a profound challenge to global health. Based on findings from the World Heart Federation (WHF) Global COVID-19 Study, an international prospective cohort study, this editorial reflects on the enduring burden of symptoms and complications among 2,535 previously hospitalized patients across 16 countries during the Omicron era. Beyond a mortality rate of 15% and clinical manifestations such as fatigue, dyspnea, and adverse cardiovascular events, the study highlighted substantial psychosocial and socioeconomic impacts, with reduced work capacity and functional limitations particularly affecting populations in low- and middle-income countries captured through EuroQol 5-dimension scale and employment data. These findings emphasize that the burden of Long COVID extends beyond individual health, with significant implications for healthcare systems and economic stability. Addressing this challenge requires ongoing multidisciplinary research, validated diagnostic criteria, novel biomarkers, and effective preventive and therapeutic strategies. Furthermore, decentralized monitoring models—exemplified by telephone-based data collection in the WHF study—may offer scalable approaches to improve surveillance and inform global health policies for current and future public health crises.

## Background

The public health crisis of the COVID-19 pandemic has ended, particularly due to mass vaccination worldwide. However, infection by new strains of SARS-CoV-2 is an ongoing reality and its potential long-term consequences (also known as ‘Long COVID’) continue to raise interest in the scientific community (1,2). The 2024 National Academies of Sciences, Engineering, and Medicine definition of Long COVID states that ‘it is an infection-associated chronic condition that occurs after SARS-CoV-2 infection and is present for at least 3 months as a continuous, relapsing and remitting, or progressive disease state that affects one or more organ systems’ (3). Estimates of Long COVID incidence vary widely, ranging from 50–85% among unvaccinated individuals who were hospitalized, 10–35% among unvaccinated non-hospitalized individuals, and 8–12% in vaccinated persons (4). Understanding the burden and determinants of Long COVID is crucial to properly diagnose, develop preventive strategies, and evaluate the resources needed to address this condition.

The World Heart Federation (WHF) has previously conducted a study during the earlier phases of the COVID-19 pandemic, providing insights into the short-term cardiovascular manifestations and risk factors in hospitalized patients (5). In this context, this new WHF study expands the knowledge by presenting meaningful findings into the long-term outcomes of hospitalized COVID-19 patients, specifically within the evolving landscape of the Omicron variant and increased vaccination coverage (6).

## The WHF Global COVID-19 study

The WHF Global COVID-19 study (6), an international, prospective cohort study, followed 2.535 patients hospitalized with COVID-19 between January 2022 and August 2023 in 16 countries for up to one year after discharge, aiming to characterize short (1 and 3 month), medium (6 month) and long-term (9–12 month) outcomes, including functional, psychosocial impacts, persistent symptoms, rehospitalization, mortality, and to explore their associations with clinical and demographic predictors. At the baseline, this cohort consisted predominantly of middle-aged individuals (mean age 59.5 ± 20 years), with 55.5% being male and over half having at least one cardiovascular comorbidity. Over 63% resided in low- and middle-income countries (LMICs) and 77% were of Asian ethnicity. Overall, 89.1% of participants had laboratory-confirmed COVID-19, diagnosed by an antigen test or RT-PCR (6).

**Figure 1:**
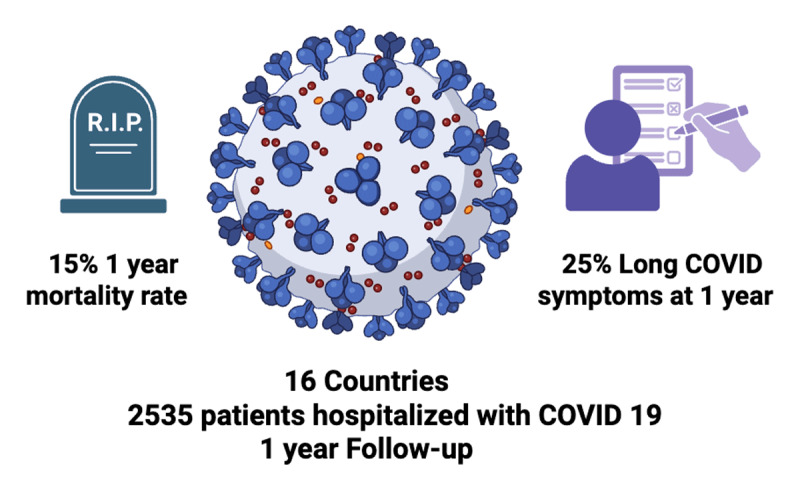
The World Heart Federation Global COVID-19 Study: Multi-centre prospective cohort.

The WHF study (6) results revealed a high prevalence of Long COVID at one month (56%), with fatigue, anxiety, and breathlessness being the most commonly reported symptoms. While the prevalence of symptoms decreased over time, a substantial proportion of patients (25%) continued to experience Long COVID at 9–12 months. Furthermore, the study identified new-onset worrying conditions such as pulmonary embolism (7.6%), kidney disease (4.0%), and hypertension (3.1%), as well as a high mortality rate of 15% (5% in-hospital and 10% during one-year follow-up period) (Figure 1). Risk factors for mortality and major adverse cardiovascular events included older age, underweight, obesity, intensive care unit admission, and pre-existing comorbidities.

The study focused on the impact of the Omicron variant, which was the predominant strain during the study period (98.2% of cases) (6). While some studies have suggested that Omicron may be associated with less severe acute illness (7,8), the WHF study demonstrates that it is associated with significant long-term morbidity and worsened prognosis (6).

These findings are consistent with a growing body of evidence underscoring the long-term impact of COVID-19 (9,10). Notably, the WHF study (6) contributes significantly by incorporating a substantial representation from LMICs with 13 out of 16 participating countries being LMICs. The burden of Long COVID in LMICs may be exacerbated by limited access to healthcare, inadequate resources, and pre-existing health disparities. Beyond the immediate health consequences, Long COVID has extensive economic and social implications. The WHF study reported that approximately 5.6% of patients experienced reduced capacity for work or mobility, often leading to changes in their employment status due to COVID-19 infection, most commonly resulting in unemployment or retirement. The EQ-5D questionnaire revealed that 39% of participants reported difficulties in performing usual activities at 1 month with a gradual decline to 17% at 9–12 month follow-up. Impairments in other domains were also prevalent, particularly early after discharge. This loss of productivity and income can have a huge effect on individuals and their families, contributing to a broader socioeconomic disruption. Moreover, this additional burden may exacerbate the strain on already overstretched healthcare systems (11).

The vaccination status has been shown to have a potential protective effect against the development and persistence of Long COVID and its prognosis. In the WHF (6) cohort, a 66.6% vaccination rate was reported at hospital admission. Patients with two or more vaccine doses prior to the SARS-CoV-2 infection have been shown in other studies to have lower rates of persistent symptoms at both 12 and 24 months after the diagnosis, compared to unvaccinated individuals (12,13), but this analysis was not thoroughly examined in this WHF study. Finally, the SARS-CoV-2 virus has evolved over the years, and the current variants may have a different impact on hard clinical outcomes or Long COVID.

The WHF study authors adopted a structured and clinically valid approach in identifying Long COVID by recording self-reported symptoms that persisted or emerged 28 days after discharge in confirmed COVID patients, a similar approach to that used by the United States of America Centers for Disease Control and Prevention (USA-CDC). Although this is a pragmatic epidemiological approach in assessing Long COVID, the lack of biomarkers or imaging modalities imposes difficulties in diagnosing Long COVID objectively. As a result, establishing a causal relationship among COVID-19, reported symptoms, and clinical events remains challenging, particularly regarding subjective manifestations. Further research is required to identify and validate objective biomarkers and imaging alterations that are specific to the diagnosis of Long COVID-19. In fact, multi-omics research has identified potential biomarkers that could elucidate the pathogenesis of Long COVID, which could lead to diagnostic strategies (14).

Of note, data collection via telephone introduces difficulties in evaluating subjective complaints. While potentially limited, decentralized models of patient monitoring demonstrate that meaningful longitudinal data can be captured beyond traditional in-person infrastructures. These models leverage accessible technology to facilitate continuous health monitoring, offering a pragmatic approach to data collection. This low-cost surveillance system has the potential to be both feasible and scalable, particularly in LMICs where healthcare resources and infrastructure may be limited. Implementing such systems can improve surveillance, enable early detection of health trends, and support timely interventions. Moreover, these models can enhance patient willingness to participate in clinical studies along with more active participation in their own health management. When integrated into broader healthcare strategies, decentralized monitoring can contribute significantly to improving health outcomes, especially during public health crises or in remote and underserved communities.

Some other limitations should be acknowledged. The WHF study (6) only tracked individuals hospitalized with COVID-19 for up to one year; hence, the results may not apply to less severe cases and longer-term cohorts demonstrated that symptoms and complications related to Long COVID may persist beyond this timeframe, with symptoms such as fatigue, amnesia, and concentration difficulties, impacting quality of life and reinforcing the hypothesis of sustained prolonged morbidity (15). Finally, given that the WHF cohort data are predominantly derived from Southeast Asia and the Asia-Pacific regions, the results of the study may not be generalizable to other regions due to environmental or genetic differences that may result in the heterogeneity of the long-term effects of COVID-19 across different populations.

## Conclusion

Overall, the WHF Global COVID-19 Study provides valuable insights into the burden and potential determinants of Long COVID in a diverse population who were previously hospitalized with a confirmed COVID-19 Omicron variant infection. It also emphasizes the potential for generating high-quality research through decentralized models. This important study should encourage further research to enable ongoing assessment across current variants, inform the development of more precise diagnostic criteria for Long COVID, and evaluate the effectiveness of interventions aimed at reducing the burden on patients.
